# A Framework for a New Approach to Empower Users Through Low-Cost and Do-It-Yourself Assistive Technology

**DOI:** 10.3390/ijerph18063039

**Published:** 2021-03-16

**Authors:** Thais Pousada García, Jessica Garabal-Barbeira, Patricia Porto Trillo, Olalla Vilar Figueira, Cristina Novo Díaz, Javier Pereira Loureiro

**Affiliations:** 1 CITIC, TALIONIS Research Group, Universidade da Coruña, 15071 A Coruña, Spain; javier.pereira@udc.es; 2 AGAELA (Galician Association of Amyotrophic Lateral Sclerosis), 15670 A Coruña, Spain; jessicagarabal@gmail.com; 3 COGAMI (Galician Confederation of People with Disabilities), 15704 Santiago de Compostela, Spain; cegadi@cogami.gal (P.P.T.); cegadi@cogami.es (O.V.F.); crd.fingoi.terapiaocupacional@cogami.gal (C.N.D.)

**Keywords:** framework, do-it-yourself (DIY), occupational therapy (OT), assistive technology (AT), design, low-cost, outcome measures

## Abstract

Background: Assistive Technology (AT) refers to “assistive products and related systems and services developed for people to maintain or improve functioning and thereby to promote well-being”. Improving the process of design and creation of assistive products is an important step towards strengthening AT provision. Purpose: (1) to present a framework for designing and creating Low-Cost AT; (2) to display the preliminary results and evidence derived from applying the framework. Methodology: First, an evidence-based process was applied to develop and conceptualize the framework. Then, a pilot project to validate the framework was carried out. The sample was formed by 11 people with disabilities. The measure instruments were specific questionnaire, several forms of the Matching Person-Technology model, the Psychosocial Impact of Assistive Device Scale, and a tool to assess the usability and universal design of AT. Results: The framework integrates three phases: Identification (Design), Creation (Making the prototype), and Implementation (Outcome Measures), based on the principles of Design Thinking, and with a user-centered perspective. The preliminary results showed the coherence of the entire process and its applicability. The matching between person and device was high, representing the importance of involving the user in the design and selection of AT. Conclusions: The framework is a guide for professionals and users to apply a Low-Cost and Do-It-Yourself perspective to the provision of AT. It highlights the importance of monitoring the entire procedure and measuring the effects, by applying the outcome measures.

## 1. Introduction

Assistive Technology (AT) refers to “assistive products and related systems and services developed for people to maintain or improve functioning and thereby to promote well-being” (World Health Organization, 2017). “It enables people with difficulties in functioning to live healthy, productive, independent and dignified lives, participating in education, the labour market and social life,” being essential tools [1]. The number of people with disabilities or elderly people that experience difficulties performing independently the Activities of Daily Living (ADL) is increasing. The World Health Organization (WHO) estimates that more than 1 billion people in the world need some form of AT. However, only 1 person in 10 has access to the devices that he or she needs [1]. 

While there is renewed emphasis on access and unmet needs, access to products is not the only challenge. Often, products need to be adapted to individual needs, but are often generic and fail to meet users’ specific, complex, and changing personal conditions, leading to abandonment or non-use [2]. Moreover, two individuals with the same health condition can have different skills, contexts, and realities, so the AT that they need will also be different. The generic designs of assistive technology devices that are commercially produced fail because they do not meet users’ specific, complex, and changing personal conditions. This can lead to the adoption of devices with problems of usability and universal design [3]. On the other hand, the broad diversity of environments in which AT is needed could complicate the process of commercial design and its provision. Various studies have shown that the current systems to obtain any assistive device do not adequately consider the contexts or environments in which the AT will be used [4,5,6]. Besides, although some models and taxonomies tend to guide the selection and the prescription of assistive technology [7,8,9], none of them takes into account the process of designing and/or creating the Low-Cost AT, involving and empowering the user during this procedure, working together with the professionals, and highlighting the user-centered design. 

What we argued previously implies more emphasis on how products can be adapted to individuals is needed. Thus, it is vital to create resources and devices that will promote the autonomy, social participation and quality of life of people with disabilities [10]. Promoting new models with this perspective and incorporating new evidence and the possibility of Low-Cost materials and 3D printing into the AT is a needed step in the field [10].

For professionals, such as occupational therapists, speech therapists, physiotherapists, and rehabilitation physicians, the selection of any AT is not an easy task, because it is necessary to consider different factors related to the user, the characteristics of the device itself, and the context in which the activity is performed [11]. As such, the prescription of any AT must be a systematic process, which implies a multidisciplinary and user-centered approach, and which includes methods for the documentation and assessment of the whole procedure [12].

In this sense, the assistive devices will be totally integrated into the life of a person and will be a guarantee of effective solutions if they are conceptualized under the guidelines of a consensus model. So, to facilitate that process, different models or frameworks have been proposed for the selection of assistive technology, including descriptive and/or predictive functions that support the provision of AT and its application in daily living [10,11,12]. Those models tend to find an optimal interaction between the person, the context, and the device, proposing valid predictions about which users could benefit from concrete devices, and identifying opportunities to increase the efficacy of the AT in practice [13]. 

The term ‘Low-Cost’ applied to the field of AT can be understood as a philosophy that includes the search, design, and creation of different objects that have been elaborated specifically for the particular needs of a person, or those that are already on the market, but always with a minimal investment [14]. Thus, Low-Cost implies that the AT satisfies, effectively and economically, the needs detected by a user in his/her Activities of Daily Living (ADL) and other important activities. On the other hand, the term ‘Do-It-Yourself’ (DIY) applied to assistive technology refers to the creation and adaptation of a device by ‘non-professionals or amateurs’, including people with disabilities and their families [15]. DIY is a movement that advocates the creation, modification, or repair/recycling of objects to meet individual criteria and have a unique design [16].

The customization of devices, based on the Low-Cost perspective and the creative design, is a practice that facilitates the creation of products potentially usable and totally adapted to the needs and abilities of one person. So, the development of customized AT from the perspective of Low-Cost contributes to the fact that it is ‘iterated in situ’, and therefore, that it can be updated and adapted in response to the user’s situation, at all times [14,15].

Different studies and resources have shown that the application of Low-Cost solutions and the development of products based on the DIY philosophy are a reality [15,17]. Nevertheless, for these proposals to be fully implemented and guarantee effective solutions, they must be carried out under the umbrella of a guidance framework.

With this in mind, the broad perspective about the application of Low-Cost (effective and economic solutions) and Do-It-Yourself (DIY) practice in the provision of AT is the baseline of this paper.

Aims: (1) to present a protocol to design and create AT from the perspective of Low-Cost and Do-It-Yourself, including outcome measures; (2) to show the preliminary results derived from applying the framework, determining the impact of AT on the life of people with disabilities.

## 2. Materials and Methods

### 2.1. Study Design

To develop the provisional framework, the research group drew on the current evidence, user and professional experiences, and design-thinking methods. To refine and test the utility of the framework, a pilot study, with a longitudinal perspective, was carried out. Also, we used outcome measures tools to determine the impact of AT on the lives of persons with disabilities.

### 2.2. Period of Study and Context

The study was carried out in the region of Galicia (Spain), led by a research group from the University of A Coruña. The fieldwork was done in the facilities of various non-government organizations (NGO) of that region that provide services to people with disabilities. That fieldwork was carried out by researchers and the collaborators’ professionals (occupational therapists) of the NGOs.

The study started in April 2019, and it is still running in order to complete 24 months. As such, the results showed in the present manuscript are preliminaries. 

### 2.3. Sample and Recruitment of Participants

The following inclusion and exclusion criteria were used:

- Inclusion criteria (met all of them):

1. Persons who have any type of disability, recognized by the correspondent certification (according to the Spanish Law of Disability—R.D. 1971/1999).
2. Persons who have a disability derived from a neurological injury (brain injury, spinal cord injury, multiple sclerosis) or from a rare disease (according to Orpha.net, a disease is considered to be rare when it affects 1 person per 2000).
3. Having reduced personal mobility and/or difficulties in the performance of ADL.
4. Receiving services or intervention from any of the NGOs’ collaborators.

These NGO collaborators attend to people of all ages; therefore, age is not a criterion for participating in the study. In the case that any participant is older than 12 and younger than 18, an adapted information sheet is given, and the informed consent is also asked of his/her legal representatives. In the case of participants younger than 12, the information sheet and informed consent were given by legal representatives.

- Exclusion criteria:

1. Persons that after reading the information sheet, do not give their informed consent to participate in the study.
2. Persons with disabilities that do not require, or do not feel the need of, improvement in their performance or independence in ADL.

The recruitment of participants was done by the occupational therapists of the NGO. In all cases, participation in the study is offered to people who meet the inclusion criteria and are given the information sheet and the document of informed consent asking for their authorization to carry on the complete procedure. 

### 2.4. Procedure

The development and conceptualization of the framework was an evidence based process, considering the previous research on the topic, the experience of professionals and research group, and the user experience. The process of creating a new AT implies implementing a systematic and user-centered methodology.

- Systematic review: To obtain good background information about the topic of the study, the PRISMA statement was applied to conduct a review of the literature. The databases consulted were Medline, Scopus, Web of Science, Cinahl, and Science Direct, using the keywords: assistive technology, Low-Cost, Do-It-Yourself, Design, 3D Printing, Framework. After applying the filters, the selected manuscripts were read completely, extracting the key concepts and the important specifications to build the base of our framework.
- Professionals’ experience: The perspective of the professionals working in the field is vital to propose a methodology that would finally be implemented to guide the process of creating Low-Cost AT. This step involved several meetings with the professionals involved (occupational therapists, physiotherapists, and informatics engineers), applying techniques of brainstorming and write-storming, followed by an offline task, in which each professional had the opportunity to review and make contributions, modelling the final framework.
- User’s experience: The process of creating an assistive device must involve the final user (person with a disability and his/her family), because otherwise there is a risk that the proposed solution does not respond to the demands, priorities and needs of the individual. Based on previous research [13], [14], [18,19,20,21], the key factors were selected in order to be incorporated into the framework. After that, several non-structured interviews with different profiles of people with disabilities were carried out to complete the general perspective of the user. After a qualitative analysis of their speeches, the significant impressions and viewpoints were incorporated into the conceptualization of the framework.

Finally, the framework for designing and creating Low-Cost Assistive Technology was conceptualized and based on the ‘Design Thinking’ method [22]. The main stages of this process (empathize, define, ideate, prototype, and test) were grouped into three phases in the present framework (according to findings of the systematic review, professionals’ and users’ experience)—Identification (design), Creation (making the prototype) and Implementation (outcome measures)—which are presented and described in the Results section. That perspective facilitates the generation of ideas and solutions, where the person with a disability has the main role. He/she is involved from the beginning, giving priority to his/her opinions, preferences, and needs.

This framework was, finally, revised by an experts’ committee in order to determine its structure, coherence, and adequacy. That committee was formed by two professionals, two users and two members of the research group, and, after a discussion meeting, gave their feedback about the framework through a consensual report. Appendix A reflects this procedure to conceptualize the framework.

### 2.5. Variables and Measuring Instruments

The main independent variable addressed in this framework is the psychosocial impact of Low-Cost AT on the quality of life of people with disabilities. That characteristic is assessed by the Psychosocial Impact of Assistive Device Scale (PIADS). The PIADS “is a 26-item, self-report questionnaire designed to assess the effects of an assistive device on functional independence, well-being, and quality of life”. The instrument includes three dimensions or sub-scales: competence (feelings of competence and efficacy—12 items), adaptability (willingness to try out new things and to take risks—6 items) and self-esteem (feelings of emotional health and happiness—8 items) [23].

The rest of the variables and measurement instruments are described below:

- Sociodemographic characteristics: Research group designed a brief questionnaire to obtain data about age, gender, marital status, the nucleus of coexistence, place of residence, diagnosis or health status and center of intervention.
- Priorities and needs of the user in vital areas: Through an interview with the final user of the Low-Cost AT, the research group applies the Initial Work Sheet from the Matching Person and Technology (MPT) Model [24]. This questionnaire makes it possible to collect information about limitations and strengths that the person has in the specified domains (thinking-understanding, seeing, hearing, speech and communication, reading and writing, mobility, dexterity/hand use, self-care, domestic life, interpersonal interactions, employment, recreation/leisure). Also, for each domain, the user can indicate his/her goal and the AT most adequate to mitigating the limitation.
- Level of user’s satisfaction in different areas: This variable is collected with the Assistive Technology Device Predisposition Assessment (ATD PA)—section A, from the MTP Model [24]. The questionnaire aims to obtain quantitative data about the subjective health perception of the participant. The 12 items of this form can be scored with a Likert scale from 1 (not satisfied) to 5 (very satisfied).
- Level of matching between person and technology: This is an outcome measure variable, which is assessed with the ATD PA—Device Form, from the MPT Model [24]. It makes it possible to determine the match between the user and his/her AT (in this case, the Low-Cost AT) through 12 items, concerning the level of adaptation to the AT in different daily situations. The user can score each item with a Likert scale from 1 (almost never) to 5 (almost always).

Moreover, the complete framework contemplates the assessment of two variables related to the assistive device itself:

- Usability: This concept is defined as “the extent to which a product can be used by specified users to achieve specified goals with effectiveness, efficiency, and satisfaction in a specified context of use”. Usability also refers to methods for improving ease-of-use during the design process [25]. The procedure of creating Low-Cost AT includes the evaluation of usability characteristics of each device, applying the System Usability Scale. This tool makes it possible to assess the level of usability of any object or device, including 10 questions that allow the user to score, on a Likert scale (from 1 to 5), different concerns related to the use and functional characteristics of the product itself [26].
- Universal design: This is defined as “an environment that allows the AT to be accessed, understood and used to the greatest extent possible by all people regardless of their age, size, ability or disability” [27]. So, any device should be designed to meet the needs of all people who wish to use it. For instance, our framework includes an assessment of the principles of universal design: Equitable Use, flexibility, simple and intuitive, perceptible information, tolerance of error, low physical effort, size and space for approach and use [28]. With this in mind, the research group created a self-made questionnaire, to assess and score the compliance of the Low-Cost AT with regards to each of the 7 items of Universal Design on a Likert Scale from 1 (nothing or almost nothing) to 5 (much).

### 2.6. Data Analysis

The descriptive analysis includes the expression of Mean (M), Standard Deviation (SD), and range (minimum-maximum) for the quantitative variables, and the frequencies and percentages for the qualitative ones.

To determine possible relationships and causalities between variables, some inferential analyses were done. The Kolmogorov–Smirnov test is applied to determine the normal distribution of the sample and to determine the type of test used in inferential analyses. The association between quantitative variables is checked by the Pearson correlation or Rho Spearman test. For qualitative variables, the ꭕsquare is used, or the likelihood ratio, if the observed frequencies are lower than 5%. In the case of association of quantitative and qualitative variables, the mean comparison is done with a t-student paired test or the U Mann Whitney test, according to the distribution of the sample.

The statistical analysis is done with the software SPSS v.24^®^ for Windows 10 (IBM SPSS Statistic 24, New York, NY, United States), and the signification of the hypothesis contrast is established at 5%.

### 2.7. Ethics Concerns

This study meets the current ethical standards that regulate research with people, such as the Helsinki Declaration, the Oviedo Convention on Human Rights and Biomedicine, the Regulation (EU) 2016/679 of the European Parliament and of the Council of 27 April 2016 on the protection of natural persons concerning to the processing of personal data and the free movement of such data, as well as the specific Spanish laws that protect the autonomy of the patient and the right to information.

All participants receive an information sheet with the complete description of the characteristics of the research and can sign the informed consent after reading the document.

All gathered data is previously codified, and the research group uses the REDCap (Research Electronic Data Capture) for the data collection. REDCap is a secure, web-based software platform designed to support data capture for research studies, providing: (1) an intuitive interface for validated data capture; (2) audit trails for tracking data manipulation and export procedures; (3) automated export procedures for seamless data downloads to common statistical packages; and (4) procedures for data integration and interoperability with external sources [29].

This study has the approval of the Galician Ethics Committee of Research with the reference number: 2019/215.

## 3. Results

### 3.1. A Framework for Designing and Creating Low-Cost AT

The main result of this manuscript is the complete framework for guiding the process of designing and creating Low-Cost AT for/with people with disabilities. In this section, the defined framework is presented, as well as the preliminary findings from its application.

As mentioned before, the framework is based on the methodology of Design Thinking. That perspective facilitates the development and planning of ideas and solutions in which the user is the vital part of the process, implicating him/herself from the beginning and giving priority to his/her opinions, preferences, and needs. The process of Design Thinking is integrated into 5 stages that do not have to be consecutive, being able to advance or go back according to the information that the developer is obtaining (empathize, define, ideate, prototype, and test) [22], [30].

After the procedure explained in the methodology section, the research group conceptualized a framework, understood as a circular process, in which the user is the central axis. The process is condensed in three stages:

- Phase 1—Identification (Design): This stage includes the Exploration of users’ needs and the identification of those that could be met through the use of Low-Cost AT. Moreover, it contemplates the exploration of the design and the generation of different solutions that could meet the specific needs detected. It aims to identify and understand the problem, as well as to clearly define the main goal for the user: What need is it intended to satisfy?

In this phase, standardized and quantitative tools are used to gather the data from the user and family, but also non-standardized methods. The first ones include a specific questionnaire (explained in the methodology section), the Initial Worksheet for the Matching Person & Technology Model (MPT) and the assistive technology device predisposition assessment (ATD PA)—section A are used. Concerning non-standardised tools, the professionals can use different resources, such as group discussion meetings (in which users, relatives and healthcare professionals all participate), personal and non-structured interviews with the users and their families, home visits, and the observation of user performance in various activities in his/her natural environment, among others. The last step of this phase is the establishment of basic requirements to be met by the Low-Cost AT. All collected data and relevant information are reflected in a summary table that helps to establish priorities (Table 1).

- Phase 2—Creation (Making the prototype): This stage aims to elaborate the first sample (or prototype) of the Low-Cost AT, which makes it possible to check whether the initial idea of the design effectively and adequately meets the demands of the user. The prototype must be created with the user and family, considering several factors: Universal Design principles, the activity for which the device would be used and the environment in which it would be used, maximum adaptation to the dimensions of the final product, safety and stability requirements, and adequate test performance. The prototype can be created using Low-Cost materials (such as foam, suction cups, Velcro, wires, cable ties and cardboard), or printed with a 3D printer (before the design of the device with specific software).

In this stage, the tools to quantify the design are the System Usability Scale [26] and the Checklist of the fulfillment of Universal Design Principles, explained before. The testing of the device into the user’s context and the real situation will facilitate the definitive design and the tuning of the AT. Also, the need for training in its use and its maintenance must be specified in this phase.

- Phase 3—Implementation (Outcome measures): Here, the incorporation of the final Low-Cost AT into the daily life of the user, during the performance of that activity for which it was designed, is contemplated. It makes it possible to obtain direct feedback from the user and his/her family and to detect possible failures in order to make the corresponding modifications.

**Figure 1:**
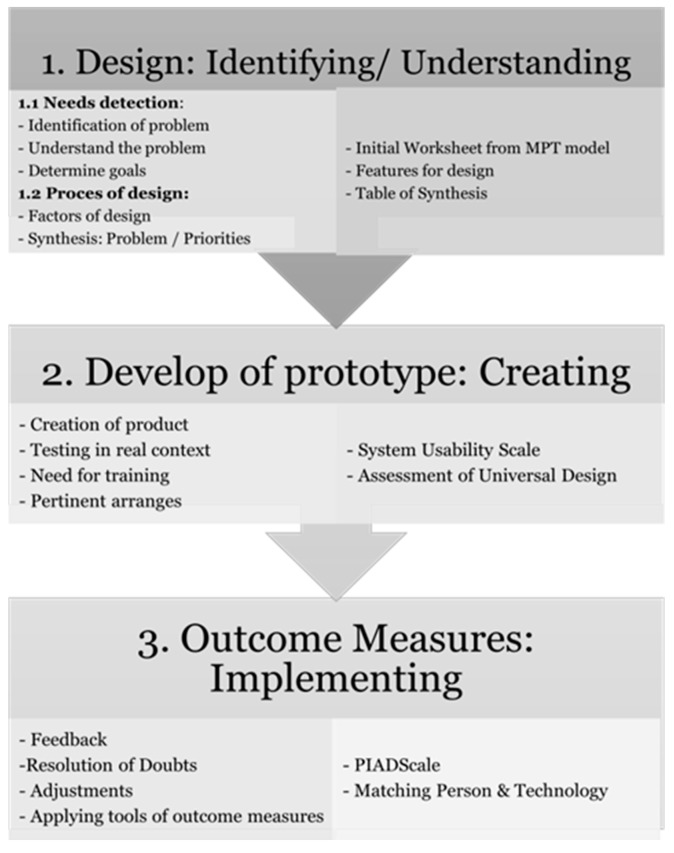
Global structure of the framework.

This phase implies the application of outcome measure instruments that makes it possible to quantify the real impact and match of the AT in the life of the user. The framework proposes the use of two of these tools: The Psychosocial Impact of Assistive Device Scale (PIADS) [23] and the Assistive Technology Device Predisposition Assessment (ATD PA), a device form of the MPT model [31]. Figure 1 summarizes the global structure of this framework.

### 3.2. Preliminary Results

In this section, the first results derived from the application of the framework are presented, in order to give a global perspective of its adequacy in the field and, ultimately, to show its efficacy and coherence.

The sample of participants in this preliminary report was formed by 11 persons with disabilities, aged from 7 to 52, 54.5% of whom were men and 45.5%, women. The diagnoses were grouped into 5 categories: Neuromuscular Disorders (NMD, including muscular dystrophies, Amyotrophic Lateral Sclerosis and myopathies), Cerebral Palsy (CP), Brain Injury, Developmental Delay and Amputations. It should be noted that the same user received several support products, so although the sample was 11 users, in fact, a total of 27 AT were created and assessed during the phases of creation and implementation.

**Figure 2:**
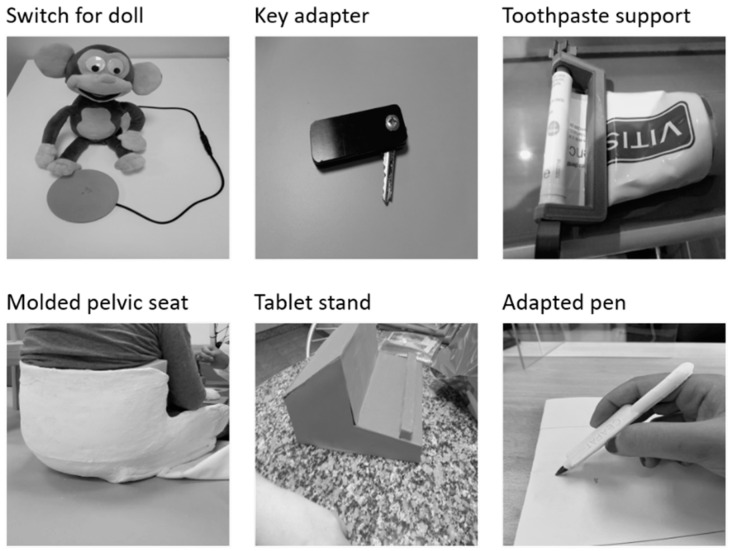
Low-Cost Assistive Technology created.

Table 2 shows the main characteristics of the sample, with the Low-Cost AT created and the participants’ perceptions of satisfaction in different vital areas (questionnaire ATD PA–MPT model). Concerning this variable, it is noted that the three most important items marked by each participant were his/her overall health (*n* = 7), autonomy and self-determination (*n* = 4), and emotional well-being (*n* = 4). Figure 2 shows several Low-Cost AT created in the research for the participants.

Concerning the priorities and needs of the participants in vital areas, assessed with Work Initial Sheet of MPT, the main domains in which people had difficulties were mobility (*n* = 7), self-care (*n* = 7), and manual skills and use of hands (*n* = 6). In this sense, the main limitations were related to walking, body balance, lack of muscular strength and difficulty in bimanual coordination, and decreased independence in bathing and grooming.

Analyzing the characteristics of the AT design process during phase 2 (a total of 27 devices), and the outcomes measure after its implementation (phase 3), the results indicate a good rating in the quality of its design (according to the principles of universal design and usability), and the matching between person and technology. In concrete terms, and with respect to the level of matching between person and technology, the item most important for the participants was “the Low-Cost AT benefits me and improves my quality of life” (indicated for 17 AT), followed by “the device fits well with my accustomed routine” (indicated for 12 AT). In contrast, the score in the subscales of PIADS (competence, adaptability and self-esteem) is low, so the impact of the Low-Cost assistive device on the lives of participants could be considered moderate. These results are reflected in Table 3. 

It is established that the AT that has obtained the highest score for design were the adapted pen and the handle for a pen (highest score in universal design), and, for the usability, the handle for pen, crutch support, key adapter and toothpaste adapter (SUS = 92.5). These results coincide with the score obtained for the degree of matching between the device and the technology, the best devices being the handle for pen, key adapter and toothpaste adapter (M = 5). The data are congruent with the score obtained in the three subscales of PIADS, where competence was the dimension with the highest score.

To detect possible correlations and influences between variables, inferential analyses were implemented. In this case, the results obtained from the scales were combined both with each other and with the sociodemographic variables. 

Demographic characteristics, such as gender and age did not have any influence on the score of outcome measures variables. The satisfaction in different vital areas is only associated with the type of diagnosis (*p* < 0.05), where the people with a diagnosis of NMD (M = 3.51), brain injury (M = 3.5), and amputation (M = 4) gave higher scores for their satisfaction with vital areas, in comparison with the person with CP (M = 2) or developmental delay (M = 2.62). 

Table 4 confirms the previous simple analysis with respect to the best ATs created. The matching between person and device is strongly influenced by the usability of the AT (*p* < 0.01), and it is also related to competence (*p* < 0.01) and adaptability (*p* < 0.05). In the case of the competence obtained by the device, it appeared to be clearly determined by the usability and universal design of the Low-Cost AT (*p* < 0.05). Self-esteem is related to the universal design of the device (*p* < 0.05), but not to the usability, while it appears that the impact of the assistive product on adaptability is not related to its design.

With respect to the type of AT, only the adaptability (subscale of PIADS) appeared to be influenced by this variable (*p* < 0.05), whereas devices for self-care were less impactful (M = 0.17), in comparison with devices for communication (M = 0.61).

The analysis of the feasibility of PIADS and ATD PA Device Form, assessed through Cronbach’s α, was moderately high (α = 0.8 and α = 0.703, respectively).

## 4. Discussion

The present work has presented a complete framework for guiding the development of AT, employing a user-centered approach and applying the base criteria of the Low-Cost and DIY perspectives. The conceptualization of this framework has followed the recommendations for the field of AT design, based on the development and the implementation of a systematic and user-centered method [32,33,34].

A pilot project has been done to get a first feedback from the implementation of the framework, as well as to determine their viability and cost-benefit. The customization of AT, based on Low-Cost, DIY and creative design, promotes the making of devices that are usable and adaptable to users [14]. 

According to the study of Philips and Zao [35], different factors (both positive and negative) have been identified as contributing to the outcome whereby a person stops using (or never uses) a recommended AT. The implementation of technologies and services that involve people in the design, development and adaptation of their own AT, based on the DIY approach, has a clear potential to reduce the conditions that can lead to abandonment [15,34].

Thus, it seems evident that the person involved in the whole process, with the user-centered perspective, implicates that he/she is part of the solutions that emerge. So, he/she will have engagement with the application of this solution (Low-Cost AT) in his/her activities of daily living.

A successful proposal to improve the design and creation of customized AT has been presented. The process makes it possible to effectively and efficiently meet the needs of the person while they perform diverse activities and daily chores. The user and his/her family must be the axis around which the process of developing products happens, in order to decrease the abandonment of the device. That practice makes it possible to create an emotional relationship between the person and his/her device, which can contribute to decreasing the stigma attached to AT (considering it as a useful tool), and to increase the use of it (the user is the creator of his/her AT) [34].

The majority of models for guiding the process of AT selection and prescription consider three main elements: the person or final user (and his/her functional ability), the activity or occupation (performance of what the person wants), and the environment or context (places in which the user participates) [7]. But none of them contemplates the assessment of the AT itself, in terms of usability and universal design.

Despite the enormous development and importance of these methods, their application in Spain, concretely, their use by clinical professionals, is not a common practice, nor is it confirmed as an innovation to be incorporated in AT provision systems. Thus, the national catalogue of AT [36] may become obsolete and would not contemplate the real needs of people with disabilities in all their vital areas, not just in their personal mobility.

According to previous research [10], one of the main shortcomings in this field is the low evidence of design techniques and/or established protocols that guide the process of creating AT from the perspective of DIY or Low-Cost. 

For this reason, the present manuscript represents a great step in the establishment of a structured framework that guides professionals during their intervention with the AT, centered in the users. 

The Low-Cost solutions attributed to Assistive Technology is a perspective that includes, not only the search, design and creation of different devices that have been created specifically for the particular needs of one user, but also the buying of products that are in the ordinary market (and are not marketed as AT themselves), and that can be a great solution in daily life, with a minimal investment. The cost, in terms of both materials and tools needed to create the device, must be minimal. Based on this idea, some examples could be the building of an adapted tricycle with low investment in materials, the use of an ergonomic opener marketed in a non-specialized store, or the use of a resin chair as an ‘equivalent’ for a shower chair.

To get the initial evidence and results of the implementation of this framework, a pilot study was performed. The sample was formed by 11 persons, and 27 AT were created and assessed (the same participant could receive one or more devices). The preliminary results support and corroborate the applicability and reliability of the framework for guiding the process of designing Low-Cost AT.

The perspective of the user and family was taken into account from the first moment, applying the Work Initial Sheet and assessing participants’ satisfaction in vital areas (MPT Model). The usability and universal design of the devices were assessed by the users, and the application of outcome measures was allowed to determine the matching and the impact of the Low-Cost device on the life of participants.

It must be highlighted that all participants had mainly physical disabilities, so the AT devices that support mobility, manual skills, and self-care were the most demanded. 

As in previous studies, competence was the dimension of PIADS with the highest score, and self-esteem, the lowest [20,37,38,39]. This can be related to the fact of the AT is conceptualized as an environmental factor, supporting the performance of activities of daily living, and so, promoting competence. On the other hand, the use of any AT implicates that its user must accept the new condition, so that dependence increases, and significant attributes of the device can be negative from the beginning [2]. 

The best results obtained for the AT, by type of devices, were for those which promote communication. This result is consistent with previous studies [37,40,41], from which it can be deduced that people with physical disabilities give higher value to the device that improves his/her communication and manipulative skills, over any other AT. Nevertheless, the National Health System in Spain only finances AT for mobility (locomotion), orthotics, and prostheses, leaving aside these communication devices that are so important for the autonomy of people [36]. The results that emerged in this type of study should make policymakers think about restructuring the national catalogues, including new and necessary AT (i.e., for communication) to improve the conditions and independence of people with disabilities.

According to Carneiro et al. (2015), no evidence in the literature systematizes, in a consolidated way, an analysis of the usability and the user experience of the AT [42]. In this sense, the framework presented here has aimed to integrate the principles of universal design and usability during the process of creating a Low-Cost AT. The preliminary results of the pilot study have shown a significant and positive influence of the characteristics of the AT’s design (in terms of usability and universal design) on the impact and matching between this device and its user, highlighting the importance of incorporating this factor during the process, and of applying user-centered perspective.

Participants with CP and Developmental delay indicated lower rates of satisfaction in different vital areas than users with NMD, amputations and brain injury. This could be explained by the fact that in the former cases, the questionnaires were completed with the help of the family of the participants, and from their perspective, the user’s perception about his/her well-being could have been underestimated. Those results must be considered, because the perspective of family and caregivers and their experience with the AT merit attention when outcome measure perspective is applied [43,44]. 

### 4.1. Limitations of the Study

Despite presenting interesting findings that support the benefits and efficacy of having a framework in the field of Low-Cost AT, the preliminary results must be considered with caution. The size of the sample is small, and the different types of diagnosis can represent a difficulty in the stratification of the sample and the application of inferential analysis. Related to the statistical analysis, although that makes it possible to determine the trends of dependent variables, it is not possible to ensure that the variations and relationships are due exclusively to the process performed, because there could have external variables exerting an influence that are not controlled in the study development. So, the obtained results only can be generalizable to the population from which the sample was selected [45]. 

Other limitations might be the device’s own design, in terms of security and durability. Taking into account the short period during which the Low-Cost AT was implemented, it is not possible to assess and determine its long-term adequacy. 

The conceptualization of the framework and the development of the pilot study must be understood within the cultural, political, and social context of the country where it was implemented, namely, Spain. So, in order to apply the framework and its phases, it is necessary to adapt them to the specific context, mainly in the use of assessment tools and the application of non-standardised tools in the identification phase.

### 4.2. Implications for Future Research

The WHO established and promoted the ‘Global priority research agenda for improving access to high-quality affordable assistive technology’, which aims to advocate the increase in the development of affordable and high-quality devices [1]. Further, AT needs to be prioritized by development stakeholders and governments as an essential component for inclusive sustainable development goals (SDG). So, to ensure equality and to contribute to the attainment of SDG, universal access to affordable assistive products needs to be prioritized by Member States and institutions worldwide [46].

Several questions are raised that should be a guide to research in this line [1]:

- What do industry leaders and others need to do to satisfy the needs of different income groups and/or countries (e.g., those with different GDP per capita)?
- How can the maintenance and repair costs of assistive products be kept as low as possible?
- How and to what extent can pervasive design principles and/or technological standards help to develop high-quality, Low-Cost solutions?

So, it becomes clear that the application of the principles of the Low-Cost and DIY perspectives in the process of creating AT must be one of the lines of future research, so the recommendations WHO can be fulfilled, especially those that advocate equal access to these devices.

The implications derived from the conceptualizations and application of this framework could be included:

- Improvement of provision system of Assistive Technology.
- Development of guidelines to ensure an efficient procedure and service.
- Determination of factors in the design of Low-Cost AT.
- Determination of prescription, matching, training and follow-up as a continuum procedure.

To complete the preliminary results and to obtain more evidence that would strengthen the framework, the research group will collect data and apply the outcome measures in the long term to confirm the obtained results. This includes increasing the size of the sample, and including participants with other vital situations and needs, such as people with low vision or blindness, deafness, and cognitive impairments.

## 5. Conclusions

This manuscript presented a unique framework addressed to guiding the process of designing and creating Low-Cost Assistive Technology for and with people with disabilities. The framework is conceptualized into three phases: Identification (Design), Creation (Making the prototype) and Implementation (Outcome Measures), based on the principles of Design Thinking, and applying a user-centered and active perspective.

The use of any AT is conditioned by the person’s predisposition, his/her priorities, and his/her expectations regarding the device. The active participation of the user in the entire process of design and creation of the AT promotes his/her self-management as a methodology that implies, educates, ensures equality and encourages empowerment.

The ideal process must incorporate not only the standards of cost-effectiveness but also the need to improve the independence and quality of life of people with disabilities in their activities of daily living.

The preliminary results have demonstrated the feasibility and good sense of the entire procedure, showing a concordance between the needs of the user, the design of his/her AT and the results of outcome measures. There are strong relationships between the design (in terms of usability and universal design) of Low-Cost AT and the matching and impact (especially the competence). The best-rated ATs with the greatest impact on users were the pen handle, key adapter, and toothpaste adapter, which are related to solving users’ deficit of manipulative skills. In general, the usability of created Low-Cost AT was high, and the matching between the person and his/her device also scored high.

The framework proposed here aims to meet the recommendations of the WHO [1] and to contribute to Sustainable Developmental Goals achievement, as AT can be considered both a mediator and a moderator of SDG [46].

## Figures and Tables

**Table 1:** Factors considered for the design of Low-Cost Assistive Technology.

**Synthesis Needs—Priorities of User**		
Detected difficulties	Consideration of the environment/context	Tasks/activities in which the AT is necessary
Interpersonal Interactions Mobility Dexterity/Hand Use Seeing. Hearing. Reading and Writing, Learning Domestic Life and Household Activities Self-care Health Maintenance. Recreation, Leisure and Social/Community Life Employment Thinking, Understanding, and Remembering	Home Workplace School Community places…	To comb To get up from the chair…
**Priority problem to be addressed in the intervention with Low-Cost AT**		
**Solutions proposed**		
Methods to find solutions	Chosen solution	Motive for choice
☐Brainstorming ☐Interview with user ☐Interview with family ☐Visits at home		
**Basic requirements of AT**		
Dimensions		
Weight		
Security factors		
Aesthetics/Appearance		
Complexity level		
Need of support from the others		
Need for installation/Maintenance		
Possible materials		
Total cost		

**Table 2:** Sociodemographic characteristics of the sample.

Variable		Diagnosis Group										Total	
		Neuromuscular Disorders		Cerebral Palsy		Developmental Delay		Brain Injury		Amputation			
		N	%	N	%	N	%	N	%	N	%	N	%
Gender	Men	3	40%	1	50.0%	1	50.0%	0	0%	1	100%	6	54.5%
	Women	2	60%	1	50.0%	1	50.0%	1	100%	0	0%	5	45.5%
Type of AT	Dressing	0	0%	0	0.0%	2	28.6%	0	0%	0	0%	2	7.4%
	Communication	3	21.4%	2	50.0%	1	14.3%	0	0%	0	0%	6	22.2%
	Play	0	0.0%	1	25.0%	1	14.3%	0	0%	0	0%	2	7.4%
	Mobility	2	14.3%	1	25.0%	1	14.3%	1	100%	0	0%	5	18.5%
	House management	4	28.6%	0	0%	0	0%	0	0.0%	1	100%	5	18.5%
	Self-care	4	28.6%	0	0%	0	0%	0	0%	0	0%	4	14.8%
	Rehabilitation	1	7.1%	0	0%	2	28.6%	0	0%	0	0%	3	11.1%
		Mean	SD	Mean	SD	Mean	SD	Mean	SD	Mean	SD	Mean	SD
Age		28.14	14.44	35.00	-	8.43	3.50	9.00	3.50	48.00	.	25.73	16.22
Mean of satisfaction ^1^		3.51	0.78	2.00	-	2.62	0.08	3.50	.	4.00	.	3.07	0.83
Sum satisfaction’s score ^1^		42.14	9.40	24.00	-	31.43	0.98	42.00	.	48.00	.	36.89	9.93

^1^ Level of user’s satisfaction in different vital areas collected with the Assistive Technology Device Predisposition Assessment (ATD PA)—section A, from the MTP Model.

**Table 3:** Results from the application of the measure instruments.

User	Type of Diagnoses	Low-Cost AT	Universal Design (sum)	Universal Design (mean)	Usability	Matching (mean)	Matching (sum)	PIADS Subscales		
								Competence	Adaptability	Self-Esteem
1	NMD	Adapted pen	34.00	4.86	50.00	3.46	38.00	0.33	0.33	0.38
		Handel for pen	34.00	4.86	55.00	3.91	43.00	1.33	0.67	1.00
2	DD	Identifier for shoes (R-L)	28.00	4.00	80.00	4.58	55.00	0.92	0.17	0.13
		Kneepads	23.00	3.29	65.00	3.80	38.00	0.17	0.33	0.25
3	DD	Zipper adapted	28.00	4.00	72.50	4.00	48.00	0.58	0.33	0.13
		Communication agenda	24.00	3.43	77.50	4.22	38.00	1.08	0.83	0.63
		Switch for doll	25.00	3.57	67.50	4.58	55.00	0.42	0.33	0.13
		Handling box	28.00	4.00	85.00	4.67	56.00	0.50	0.33	0.00
		Manipulative board	24.00	3.43	72.50	3.92	47.00	0.42	0.33	0.13
4	NMD	Opener	24.00	3.43	85.00	4.33	52.00	0.67	0.33	0.13
		Key adapter	30.00	4.29	92.50	5.00	50.00	1.50	0.33	1.00
		Toothpaste adapter	30.00	4.29	92.50	5.00	40.00	1.08	0.33	0.50
		Adapted nail clipper	24.00	3.43	85.00	3.83	46.00	0.50	0.17	0.13
5	NMD	Key adapter	22.00	3.14	40.00	2.11	19.00	0.00	0.00	0.00
6	NMD	Crutch support	29.00	4.14	92.50	3.67	44.00	0.25	0.17	0.25
		Toothpaste support 1	14.00	2.00	47.50	3.33	40.00	0.00	0.00	0.00
		Toothpaste support 2	26.00	3.71	77.50	4.58	55.00	0.17	0.17	0.13
		Long handle scissors	23.00	3.29	57.50	3.00	36.00	0.25	0.33	0.00
7	BI	Molded pelvic seat	22.00	3.14	90.00	4.11	37.00	0.08	0.33	0.38
8	Cerebral Palsy	Tablet stand	23.00	3.29	80.00	4.33	39.00	0.17	0.33	0.00
		Orientation agenda	25.00	3.57	72.50	4.45	49.00	0.58	0.50	0.25
		Texture domino	25.00	3.57	72.50	4.27	47.00	0.42	0.67	0.38
		Positioning board	26.00	3.71	75.00	4.33	39.00	0.25	0.50	0.00
9	NMD	Height-adjustable table legs	33.00	4.71	72.50	4.25	34.00	0.42	0.33	0.25
		Girth for use on an inclined plane	30.00	4.29	70.00	3.25	25.00	0.00	0.17	0.25
10	NMD	Handle for pen	24.00	3.43	92.50	5.00	50.00	0.92	1.00	0.63
11	Amputation	Adaptation for knife handle	29.00	4.14	77.50	3.82	42.00	0.75	0.17	0.25
Total Sample			Mean (SD)	Mean (SD)	Mean (SD)	Mean (SD)	Mean (SD)	Mean (SD)	Mean (SD)	Mean (SD)
			26.19 (4.3)	3.74 (0.61)	73.98 (14.3)	4.07 (0.66)	43.04 (8.93)	0.51 (0.41)	0.35 (0.22)	0.27 (0.28)

NMD: Neuromuscular Disorders; DD: Developmental Dealy; BI: Brain Injury.

**Table 4:** Significant correlations between quantitative variables.

		Matching	Competence	Adaptability	Self-Esteem	Universal Design	Usability
Mean of Matching ^1^	Rho	-	0.551 **	0.420 *	0.201	0.201	0.630 **
	*p* value	-	<0.01	<0.05	0.315	0.201	<0.01
Competence ^2^	Rho	0.551 **	-	0.467 *	0.527 **	0.447 *	0.428 *
	*p* value	<0.01	-	0.014	<0.01	<0.05	<0.05
Adaptability ^2^	Rho	0.420 *	0.467 *	-	0.461 *	0.075	0.100
	*p* value	<0.05	<0.05	-	<0.05	0.708	0.621
Self-esteem ^2^	Rho	0.201	0.527 **	0.461 *	-	0.449 *	0.263
	*p* value	0.315	<0.01	<0.05	-	<0.05	0.186
Universal Design ^3^	Rho	0.201	0.447 *	0.075	0.449 *	-	0.116
	*p* value	0.201	<0.05	0.708	<0.05	-	0.565
Usability ^4^	Rho	0.630 **	0.428 *	0.100	0.263	0.116	-
	*p* value	<0.01	<0.05	0.621	0.186	0.565	-

^1^ Matching is assessed with the ATD PA—Device Form, from the MPT Model; ^2^ Competence, adaptability and self-esteem are the subdimensions of PIADS; ^3^ Universal Design is assessed with a Likert scale (1–5); ^4^ Usability is assessed with System Usability Scale (SUS). * Good significance level (*p* < 0.05); ** Very good significance level (*p* < 0.01).

**Figure A1:**
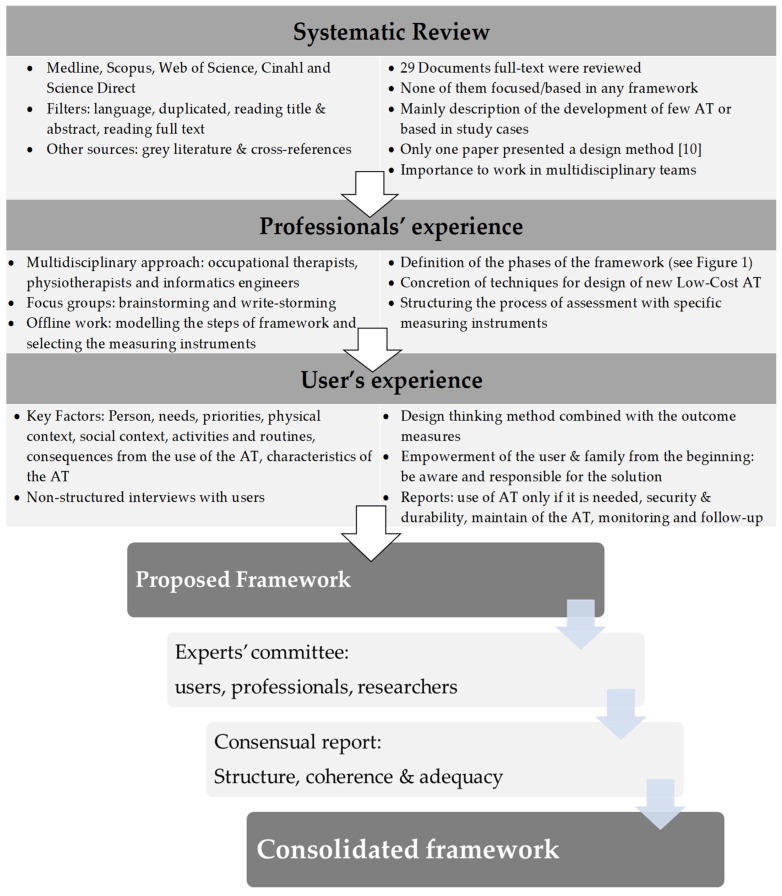
Procedure of conceptualizing the framework.

## Data Availability

Data is contained within the article.
